# NEK2 promotes oral squamous cell carcinoma progression and serves as a diagnostic and therapeutic target

**DOI:** 10.1038/s41598-026-47174-6

**Published:** 2026-04-10

**Authors:** HaiYing Wu, SiYing Huang, NingXiang Wu, WeiHua Chen

**Affiliations:** 1 The Affiliated Stomatological Hospital, Jiangxi Medical College, Nanchang University, Nanchang, China; 2 Jiangxi Provincial Key Laboratory of Oral Diseases, Nanchang, China; 3 Jiangxi Provincial Clinical Research Center for Oral Diseases, Nanchang, China; 4 Jiangxi Key Laboratory of Oncology, Department of Center Laboratory, The Third Affiliated Hospital of Nanchang University, Nanchang, China

**Keywords:** NEK2, OSCC, Cancer growth, Cancer invasion, Cancer metastasis, Targeted therapies, Cancer, Cell biology, Oncology

## Abstract

NIMA - related kinase 2 (NEK2), belonging to the NIMA family, is intricately linked to tumorigenesis and tumor progression. Nevertheless, its biological functions and underlying mechanisms in oral squamous cell carcinoma (OSCC) remain elusive. The protein expression level of NEK2 in clinical OSCC samples was evaluated using immunohistochemical staining, and its prognostic impact on OSCC patients was analyzed via Kaplan-Meier survival analysis. Additionally, RT-qPCR and Western blot were performed to examine NEK2 mRNA and protein expression levels in OSCC cell lines (SCC4 and Cal27). Scratch wound healing, Transwell, and immunofluorescence assays were conducted to assess the effects of NEK2 overexpression or knockdown on epithelial-mesenchymal transition (EMT), invasion, and migration in OSCC. Subcutaneous xenograft and tail vein pulmonary metastasis models in nude mice were further employed to elucidate the role of NEK2 in OSCC growth and metastasis in vivo. Moreover, the therapeutic effect of INH1 inhibitor on OSCC tumors was evaluated in nude mice. GO and KEGG enrichment analyses were performed to explore NEK2-regulated pathways in OSCC. The protein manifestation of NEK2 within OSCC tissues was conspicuously elevated in comparison to that in normal oral mucosal tissues. Moreover, elevated NEK2 expression was correlated with suboptimal overall survival (OS) and progression - free survival (PFS). Exhibiting the capacity to induce EMT, NEK2 consequently enhanced the migration and invasion of OSCC cells in vitro. In vivo, it promoted tumor growth, metastasis, and lung colonization. The NEK2 inhibitor, INH1, can effectively suppress the growth, metastasis, and lung colonization of OSCC in animal models. Furthermore, the evidence we have presented suggests that NEK2 may drive the development and progression of OSCC by activating the PI3K - Akt and IL − 17 signaling pathways. NEK2 holds potential as a diagnostic biomarker and a molecular therapeutic target for OSCC patients.

**Supplementary Information:**

The online version contains supplementary material available at 10.1038/s41598-026-47174-6.

## Introduction

Oral squamous cell carcinoma (OSCC) is one of the most common malignant tumors in the head and neck region, ranking sixth globally in incidence^1,2^. In recent years, its incidence has been rising, with a trend toward affecting younger populations^3,4^. Although current treatment modalities, such as surgery, radiotherapy, chemotherapy, and combination therapies, have advanced, the high rates of metastasis and recurrence result in a five-year survival rate that remains around 50%^5^, underscoring an urgent need to improve patient prognosis. Therefore, elucidating the molecular mechanisms underlying OSCC development and progression, and actively exploring early diagnostic markers and novel therapeutic targets, are of critical scientific and clinical importance for enhancing clinical management and improving patient outcomes.

Never in mitosis gene A - related kinase 2 (NEK2), a serine/threonine protein kinase associated with NIMA, is localized on human chromosome 1q32.2–1q4 and encompasses eight exons. Structurally, NEK2 possesses an N-terminal serine-threonine kinase domain and a C-terminal regulatory domain, which harbors multiple functional motifs, including centrosomal and microtubule localization sites, a protein phosphatase 1 (PP1) binding site, and a nucleolar localization signal^6^. As a multifunctional protein, NEK2 engages in diverse physiological and pathological processes through its interactions with various partner proteins. Within the centrosome, NEK2 facilitates the orderly attachment of chromosomes to spindle microtubules during mitosis, thereby safeguarding chromosomal stability and preventing mitotic errors^7,8^. However, aberrant overexpression of NEK2 induces centrosome amplification and multinucleation, leading to aneuploidy, chromosomal instability, and ultimately promoting tumorigenesis, cancer progression, and uncontrolled proliferation^9^. Elevated NEK2 expression has been documented in multiple malignancies, including hepatocellular carcinoma, lung cancer, cervical cancer, breast cancer, and colorectal carcinoma, where it correlates with enhanced tumor migration and invasiveness^10–12^. Studies have shown that NEK2 drives the proliferation, migration, and invasion of tumor cells by activating key signaling pathways such as Wnt/β-catenin^13,14^, PI3K/Akt/mTOR^15–17^, TGF-β/Smad2^18^, NIK/NF-κB^19^, and RhoA/Rac1^20^. In gastric cancer, NEK2 not only participates in regulating the cell cycle and p53-independent DNA damage response but also intervenes in glycine-mediated signaling, collectively promoting disease progression^21^. Notably, inhibition of NEK2 can upregulate the expression of the ferroptosis-related gene HMOX1, thereby enhancing the sensitivity of gastric cancer cells to ferroptosis^22^. In glioblastoma, NEK2 accelerates tumor cell proliferation and migration by promoting TP53 ubiquitination^23^. Furthermore, overexpression of NEK2 mediates resistance to multiple drugs and impairs the efficacy of PD-1/PD-L1 immunotherapy by shaping an immunosuppressive tumor microenvironment^24^.

Notably, studies have shown that NEK2 is also overexpressed in OSCC^25^, and its high expression level is significantly associated with shortened overall survival (OS) in patients^26^. Furthermore, our preliminary research demonstrated upregulated NEK2 expression in OSCC tissues, suggesting a potential link between NEK2 and the modulation of epithelial-mesenchymal transition (EMT)-related protein expression^27^. This implies that NEK2 is likely to be involved in the occurrence and development of OSCC. Nevertheless, its specific mechanism of action remains obscure.

This study aims to systematically elucidate the role of NEK2 in the progression of OSCC, and to explore its relationship with OSCC prognosis as well as its potential as a therapeutic target. First, the correlation between NEK2 expression and OSCC prognosis is analyzed. Subsequently, using a combination of in vitro and in vivo experiments, we investigate the impact of NEK2 on OSCC cell migration, invasion, tumorigenesis, and metastatic potential in vivo, while examining its association with the EMT process. Furthermore, we explore which key signaling pathways may be activated by NEK2 overexpression to exert its pro-cancer effects. Finally, the therapeutic efficacy of the NEK2-specific inhibitor INH1 against OSCC with high NEK2 expression is evaluated, thereby providing a theoretical and experimental foundation for developing novel NEK2-targeted treatment strategies.

## Materials and methods

### Cell lines and reagents

OSCC human cell lines (Cal27 and SCC4) and human normal gingival epithelial cell line (HGE) were purchased from Procell Life Science & Technology Co., Ltd. (Wuhan, China). Cal27 and HGE were cultured in Dulbecco’s modified Eagle’s medium (DMEM; Solarbio Science & Technology Co., Lld., Beijing, China) complemented by 10% fetal bovine serum (Gibco; Thermo Fisher Scientific, Inc., Waltham, MA, USA). SCC4 were cultured in Dulbecco’s modified Eagle’s medium/F12(DMEM/F12; Viva Cell; XP Biomed Co., Ltd.,Shanghai, China) complemented by 10% fetal bovine serum (Gibco; Thermo Fisher Scientific, Inc., Waltham, MA, USA). All of the cells were authenticated using short-tandem repeat profiling, tested for mycoplasma contamination, and cultured for < 2 months at 37 °C in an incubator containing 5% CO_2_.

### Plasmids

Human NEK2 was cloned using a pair of primers, which were designed based on the related sequences in GenBank through bio-software Primer5.0. The specific sequence of these primers was forward :5’-CCAGATTACGCTGAATTCATGCCTTCCCGGGCTGAG-3’ and reverse :5’-CGGATCACTAGTGCTAGCCTAGCGCATGCCCAGGATC-3’.Upon preliminary analysis with bio-software, NEK2 was cloned into pSin-HA-puro vector. All the recombinant plasmids were verified by DNA sequencing (Ruiboxingke Biotech Co., Ltd., Beijing, China).

### Antibodies

Human anti-NEK2 (cat. no. 24171-1-AP) was obtained from ProteinTech Group, Inc. The epithelial–mesenchymal transition (EMT) IF Antibody Sampler Kit (anti-E-cadherin, anti-N-cadherin, anti-Vimentin and anti-Slug) (cat. no. 49398) and anti-β-actin (cat. no. 4970) were obtained from Cell Signaling Technology.

### Stable lines

For the overexpression of NEK2 in Cal27 cells, 6.7 µg of pSin-HA-puro delivering NEK2 or 6.7 µg of the empty vector was co-transfected with 5 µg of pSa and 3.3 µg of pIG into HEK-293T cells for 48 h using Lipofectamine 8000 (Beyotime Biotechnology Co., Ltd., Shanghai, China).The recombinant viruses were subsequently collected and added to Cal27 cells for 24 h. The stable lines were selected with 1 µg/ml of puromycin for 72 h.

### RNA interference

The effective siRNAs oligonucleotide targeting NEK2 were synthesized by Shanghai Weisan Biotechnology Co., Ltd. (Shanghai, China). The cell density in each well of the 6-well culture plate reached around 80% on the day of transfection. Transfection was performed according to the manufacturer’s instructions, and the cells were transfected with 50 nM of siRNA using Lipofectamine 8000 transfection reagent (Beyotime Biotechnology Co., Ltd., Shanghai, China). The human siRNA sequences were as follows: NEK2 siRNA #1, sense 5’-GAGAAGAGGGCGACAAUUATT-3’ and antisense 5’-UAAUUGUCGCCCUCUUCUCTT-3’; NEK2 siRNA #2, sense 5’-GAGCUGAAACUGAAGGAAATT-3’ and antisense 5’-UUUCCUUCAGUUUCAGCUCTT-3’; NEK2 siRNA #3, 5’-GAAGAGUGAUGGCAAGAUATT-3’ and antisense 5’-UAUCUUGCCAUCACUCUUCTT-3’; Negative control, sense 5’-UUCUCCGAACGUGUCACGUTT-3’ and antisense 5’-ACGUGACACGUUCGGAGAATT-3’. At 48 h following transfection, qRT-PCR and Western blotting assays were performed.

### RNA extraction and qRT-PCR

Total RNA was abstract using the RNA isolater Total RNA Extraction Reagent (cat. no. R401-01, Vazyme Biotech Co., Ltd., Nanjing, China) via the Trizol method, according to the manufacturer’s instructions. cDNA was synthesized by reverse transcription using the HiScript III All-in-one RT SuperMix Perfect for qPCR (cat. no. R333-01, Vazyme Biotech Co., Ltd., Nanjing, China). qRT-PCR was performed in a CFX96 Real-Time PCR Detection system (Bio-Rad Laboratories, Inc., Hercules, CA, USA) using TB Green^®^ Premix Ex Taq™ II (cat. no. RR820A, TaKaRa Bio, Inc., Otsu, Japan). Parameters for thermal cycling included step one 94°C for 30s, step two 45 cycles of 94 °C for 4s, 58 °C for 15s and 72°C for 15s, as well as step three 65 °C for 5s and 95°C for 5s. The amplification products were analyzed with the 2^−ΔΔCq^ method and the expression level in every sample was normalized to those of the housekeeping gene GAPDH. The primer sequences were as follows: NEK2 forward (F), 5’-GATGACTCAGTTGACTCTGGC-3’ and reverse (R), 5’-TCCCGATGCAATACGGTATGA-3’; E-cadherin F, 5’-ATTTTTCCCTCGACACCCGAT-3’ and R, 5’-TCCCAGGCGTAGACCAAGA-3’; N-cadherin F, 5’-AGCTCCATTCCGACTTAGACA-3’ and R, 5’-CAGCCTGAGCACGAAGAGTG-3’; Vimentin F, 5’-GACGCCATCAACACCGAGTT-3’ and R, 5’-CTTTGTCGTTGGTTAGCTGGT-3’; GAPDH F, 5’-GAAGGTGAAGGTCGGAGTC-3’ and R, 5’-AAGATGGTGATGGGATTTC-3’. The experiments were independently repeated in triplicate.

### Western blotting

Cells were collected and lysed in a solution prepared by PMSF (Solarbio Science & Technology Co., Lld., Beijing, China) and RIPA buffer (150 mM NaCl, 0.5% EDTA, 50 mM Tris, 0.5% NP40) at 4 °C for 30 min. After centrifugating for 20 min at 14,000 rpm at 4 °C, the lysates were obtained and the Bradford method was used to determine the protein concentration. At room temperatureand, equal amounts of protein extracts were separated by 10% SDS-PAGE at a voltage of 90 V for 1.5 h, and converted to a voltage of 150 V for 0.5 h. Protein extracts were transferred to PVDF membranes with an electrical current of 350 mA at 4 °C for 90 min, and blocked with 5% non-fat milk for 1 h at room temperature. The membranes were incubated with antibodies against NEK2, E-cadherin, N-cadherin, Vimentin, Slug, β-actin overnight at 4 °C. After washing three times with PBST, the membranes were probed with secondary antibodies against rabbit (1:10,000;

401BW, Promega Corporation, Madison, WI, USA) for 1 h at room temperature and detected using an ECL chemiluminescence system (cat. no. P0018F; Beyotime, China) and exposed to radiographic film (Carestream, Catalog Number 6535876). The experiments were independently repeated in triplicate.

### Wound healing assay

Cells were cultured in 6-well plates until the confluence reached approximately 80% in each well. A scratch was made using a sterile 200 µl pipette tip in each well. After each well was washed with 1x PBS thrice, cells were incubated with serum-free DMEM or DMEM/F12 at 37 °C in an incubator containing 5% CO_2_. The wound was observed and photographed under an inverted microscope at 0 h, 24 h and 48 h and five different fields of view were selected at each time point. The size of the scratch area was analyzed using Image J software. The experiments were independently repeated in triplicate.

### Transwell assays

For the Transwell migration assay, 200 µl of serum-free DMEM or DMEM/F12 containing either 1 × 10^5^ cells was added to cell culture inserts contained an 8-µm microporous filter without an extracellular matrix coating (BD Biosciences, Franklin Lakes, NJ, USA). Then DMEM or DMEM/F12 containing 10% fetal bovine serum (Gibco; Thermo Fisher Scientific, Inc., Waltham, MA, USA) was added to the bottom chamber. After 48 h of incubation at 37 °C in 5% CO_2_, the cells on the lower surface of the filter were fixed, stained, observed and recorded under an inverted microscope. The number of migrated cells in five random optical fields (magnification, ×100) from triplicate filters was averaged using Image J software. For the Transwell invasion assay, 200 µl of serum-free DMEM or DMEM/F12 containing either 1 × 10^5^ cells was added to cell culture inserts which had an 8-µm microporous filter and were covered with Matrigel (Solarbio Science & Technology Co., Lld., Beijing, China). DMEM or DMEM/F12 containing 10% fetal bovine serum was added to the bottom chamber. After 48 h of incubation at 37 °C in 5% CO_2_, the cells on the lower surface of the filter were fixed by 4% paraformaldehyde solution for 30 min at room temperature, stained with 0.1% crystal violet staining solution for 30 min at room temperature, and used an inverted microscope to observe and record. The number of migrated cells in five random optical fields (magnification, ×100) from triplicate filters was averaged using Image J software. The experiments were independently repeated in triplicate.

### Animal experiments

All animal experiments were reviewed and approved by the Ethics Committee of the Affiliated Hospital of Stomatology of Nanchang University (Nanchang, China). All animal experiments were conducted in accordance with the ARRIVE guidelines, and this study was conducted in accordance with the guidelines approved by the Animal Platform Committee of the Biomedical Test Center of Nanchang University. Twenty female BALB/c nude mice ((Foxn1nu)^mut/mut^) ) without pathogens were purchased from GemPharmatech (Jiangsu, China). The nude mice were 4–6 weeks and had body weights of 22.5±1.0 g(mean±SEM). A total of 20 BALB/c nude mice were randomly assigned to 4 groups, with 5 mice in each group. The Cal27 cells or their derivatives were suspended in 0.1 ml PBS and injected into the tail vein of the anesthetized animals using isoflurane. For the drug treatment group, the medium (2% DMSO, 5% Tween 80, 40% PEG-300, 53% Saline, Solarbio) was injected subcutaneously, and INH1 (prepared in the medium at a concentration of 50 mg/kg, #HY-16660, MCE). Nine weeks later, the nude mice were anesthetized and euthanized via cervical dislocation. The lungs were collected, weighed, washed, fixed, and embedded in paraffin blocks, and sections were stained with H&E to confirm the drug’s inhibitory effect on metastasis. Three sections were taken from each lung sample. The number of lung metastasis nodules was counted under a dissecting microscope. The average lung colony was calculated for each lung sample’s 3 sections. For the subcutaneous tumor experiment group, 5 × 10^6^ Cal27 cells suspended in 200 µL of PBS were injected into the inguinal region of mice. Intervention was initiated on day 7 post-injection, at which time the tumor diameter was measured to be 0.7 ± 0.02 cm. Mice were measured for tumor volume every 5 days using the formula V = L X W ^2^/2, where L and W are the length and width of the tumor, respectively, and mice were euthanized on day 28. P values were obtained from ANOVA tests.

### Hematoxylin-eosin (H&E) staining

Lung tissue is fixed with 4% PFA in normal saline and embedded in paraffin. Sections (4 μm) were stained with hematoxylin and eosin and then examined with OLYMPUS BX43F(OLYMPUS, Japan) 200/400 electron microscopy.

### Immunohistochemical (IHC) and histological evaluation

The tissue sections were precisely sliced into 4-µm specimens subsequent to being embedded in paraffin. The samples underwent dewaxing and dehydration procedures at room temperature, succeeded by antigen retrieval through microwave heating in citrate buffer (pH 7.8), where 3% hydrogen peroxide solution was utilized to inactivate endogenous enzymes. Subsequently, the slides were effectively blocked by goat serum blocking solution. The tissue sections were further subjected to incubation with the primary antibody (1:200 dilution) at 4 °C for 15 h, and then washed at room temperature for 30 min. Subsequently, 100µL biotin-conjugated goat anti-mouse/rabbit IgG working solution (1:100 dilution, ZSJB-Bio) was added and incubated at 37 °C for 1 h. Eventually, the sections were incubated with DAB (3,3’-diaminobenzidine, ZSJB-Bio) for staining purposes, followed by hematoxylin counterstaining. The sections were scanned using a 3D HISTECH, Magya ország digital tissue section scanner. The staining scores were evaluated by employing the German semi-quantitative scoring method, with each specimen’s score being determined by the combined scores of the staining intensity (no staining = 0, weak positive = 1, medium positive = 2, strong positive = 3) and the proportion of positive cells (0% = 0; 1% − 25% = 1; 26% − 50% = 2; 51% − 75% = 3; 76% − 100% = 4). The final score was equivalent to the product of the staining intensity score and the proportion of positive cells score.

### Patients and follow-up

The human OSCC tissues used in this study were reviewed and approved by the Ethics Committee of the Affiliated Hospital of Stomatology of Nanchang University (Nanchang, China). Written informed consent was obtained from all patients. This part of the research was conducted in strict accordance with the Declaration of Helsinki. The study included 62 patients with primary OSCC who were admitted to the Affiliated Hospital of Stomatology of Nanchang University between September 2014 and September 2021. Inclusion criteria: pathologically confirmed oral squamous cell carcinoma; no preoperative radiotherapy, chemotherapy, or other interventional treatments. Exclusion criteria: incomplete medical records; history of preoperative radiotherapy or chemotherapy; presence of other concurrent tumors. Survival analysis was performed on the paraffin-embedded collected specimens. The mean follow-up duration was 54.5 months, ranging from 12 to 93.2 months.

### GO and KEGG enrichment assays

RNA-seq-based transcriptome profiling and bioinformatics analysis were provided by APExBIO Technology LLC (Shanghai, China). Raw sequencing data underwent quality control using FastQC, followed by filtering with fastp (https://github.com/OpenGene/fastp). The clean reads were aligned to the reference genome sequence using HISAT2. Transcript assembly was performed with StringTie, and the expression levels of all genes in each sample were calculated and normalized to TPM values. Differential expression analysis was conducted using either DESeq2 or edgeR, with genes meeting the criteria of FDR < 0.05 and |log2FC| > 1 identified as differentially expressed. Finally, relevant pathways (GO and KEGG) were identified through enrichment analysis^28–30^. All analyses were performed within the R Studio.

## Results

### In the primary tissues of OSCC, the expression level of NEK2 is elevated, and high - level expression of NEK2 portends poor prognosis for patients

**Fig. 1:**
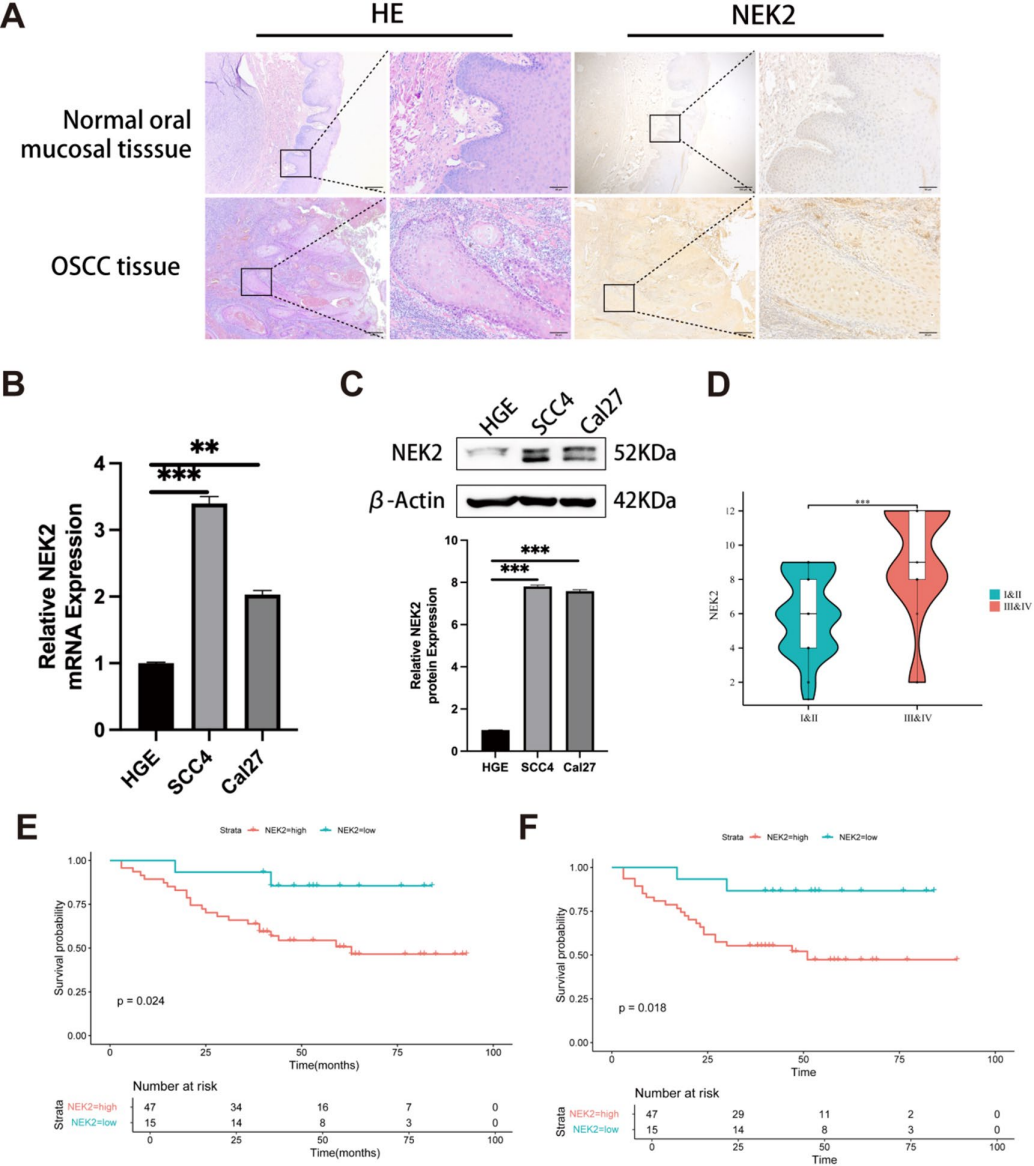
NEK2 is upregulated in OSCC and its elevated expression correlates with poor clinical outcomes. (**A**) Representative photomicrographs of H&E staining and NEK2 IHC staining in tumor tissues versus normal mucosal tissues. Scale bars, 200 μm and 50 μm, respectively. (**B**, **C**) Quantitative analysis of NEK2 expression at mRNA (**B**) and protein (**C**) levels in indicated cell lines by qRT-PCR and western blotting, respectively. GAPDH and β-Actin served as loading controls for mRNA and protein detection, respectively. Data are presented as the mean ± SD of three independent experiments. Student’s t-test, **P < 0.01, ***P < 0.001. (**D**) The protein expression of NEK2 and its correlation with clinical staging in 62 OSCC patients were meticulously evaluated through IHC. Data are presented as mean ± SD. Student’s t-test, ***P < 0.001. (**E**,**F**) OS (**E**) and PFS (**F**) curves were generated based on the NEK2 protein expression in 62 paraffin-embedded OSCC tumor tissues, *P* < 0.05.

To detect the expression of NEK2 in OSCC tissues, we performed IHC staining analysis comparing NEK2 protein levels between normal oral mucosa and primary OSCC clinical specimens. IHC staining revealed that NEK2 protein was predominantly localized in the nuclei of OSCC cells, with a minor presence observed in the cytoplasm. The staining exhibited a brownish-yellow color in the tissue sections. In contrast, NEK2 expression was absent or barely detectable in normal oral mucosal tissue cells. These findings indicate that NEK2 is highly expressed in OSCC tissues (Fig. 1A). Subsequent we utilized qRT-PCR and western blotting to assay the mRNA and protein expression levels of NEK2 in the human normal gingival epithelial cell line (HGE) and OSCC human cell lines (Cal27 and SCC4). Both Cal27 and SCC4 cells exhibited higher NEK2 mRNA and protein expression than HGE cells, with SCC4 demonstrating the most pronounced upregulation (Fig. 1B, C). These findings collectively indicate consistent NEK2 overexpression in OSCC.

To evaluate the prognostic significance of NEK2, we conducted comprehensive IHC staining and analysis on 62 OSCC tissue specimens. IHC analysis revealed that NEK2 expression was closely associated with the clinical stage of OSCC (Fig. 1D). Through receiver operating characteristic (ROC) analysis, patients were ranked according to the cutoff value of NEK2 expression and divided into two groups (the NEK2 high-expression group and the NEK2 low-expression group). Kaplan-Meier survival analysis revealed that elevated NEK2 expression significantly correlated with reduced overall survival (OS) and progression-free survival (PFS) (Fig. 1E, F). These results corroborated that the upregulation of NEK2 was closely related to the clinical stage and prognosis of OSCC and could provide clues for predicting the prognosis of OSCC patients.

### The overexpression of NEK2 facilitates the migration and invasion of OSCC cells in vitro

**Fig. 2:**
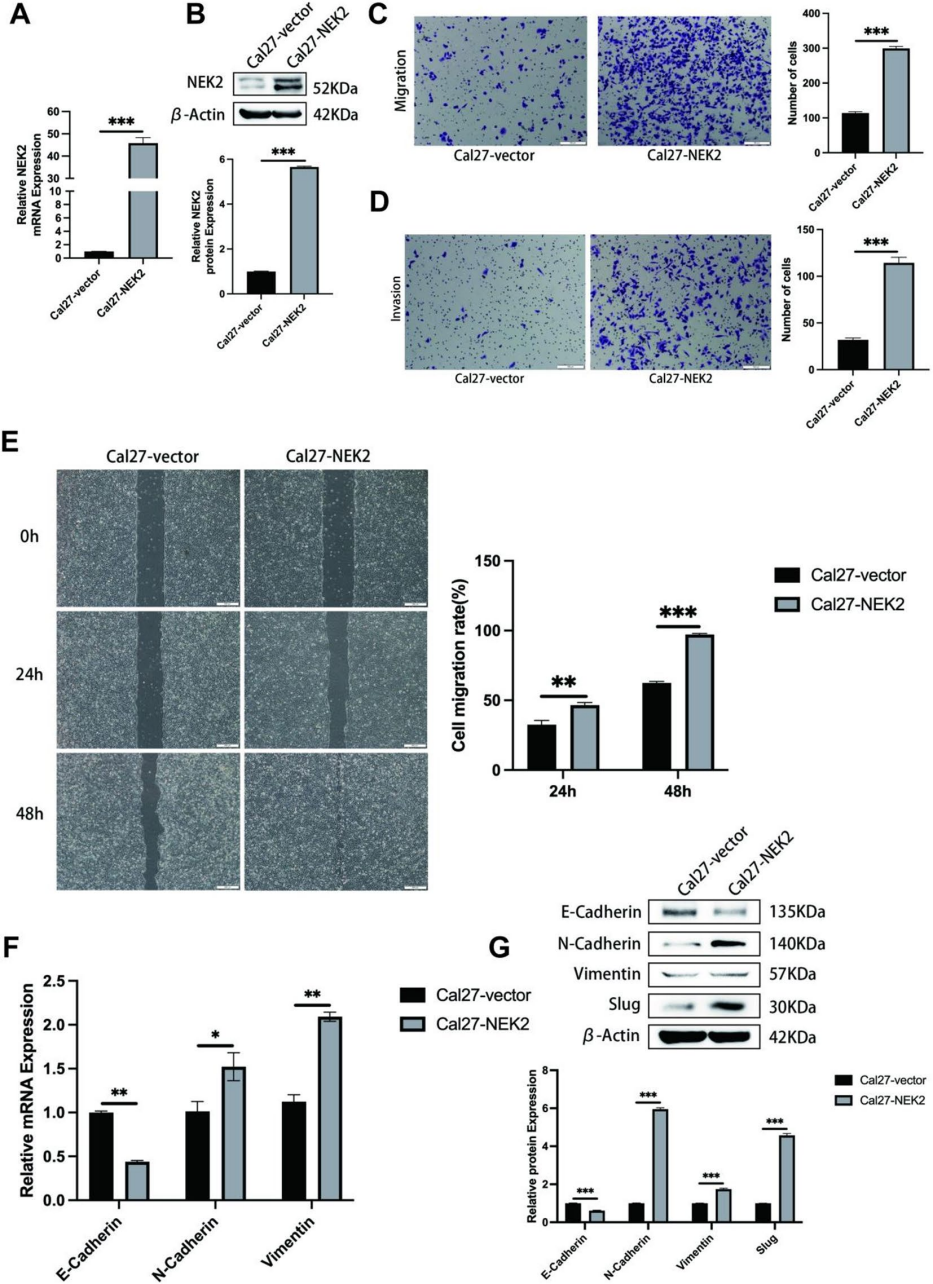
The overexpression of NEK2 facilitates the migration and invasion of OSCC cells in vitro. (**A**, **B**) The relative mRNA and protein expression levels of NEK2 in the Cal 27 cell lines stably expressing the vector or NEK2 were determined by qRT-PCR (**A**) and western blotting (**B**), respectively. GAPDH and β-Actin served as loading controls for mRNA and protein detection, respectively. (**C**, **D**) Cell migration assay (**C**) and invasion assay (**D**) were conducted in the indicated stable cell lines as described in the “Materials and methods” Section. The images on the left are representative images, and the images on the right are the statistical results. (**E**) The wound healing assay was performed in the indicated stable cell lines as described in the “Materials and methods” Section. The images on the left are representative images, and the images on the right are the statistical results. (**F**, **G**) In the indicated stable cell lines, the relative mRNA and protein expression levels of EMT-related factors were determined by qRT-PCR (**F**) and western blotting (**G**), respectively. GAPDH and β-Actin served as loading controls for mRNA and protein detection, respectively. All data represent mean ± SD from three independent experiments. Student’s t-test, *P < 0.05, **P < 0.01, ***P < 0.001.

Building upon these findings, we further investigated the role of NEK2 in OSCC. Initially, we investigated the effect of NEK2 overexpression on the migratory and invasive abilities of OSCC cells. Based on the expression of NEK2 in OSCC human cell lines (Cal27 and SCC4) (Fig. 1B, C), we constructed stably overexpressing NEK2 in Cal27 cell, which have low basal NEK2 expression (Fig. 2A, B). Results from the wound healing assay and Transwell assay, including the migration assay with cells cultured on non-Matrigel-coated membranes and the invasion assay with cells cultured on Matrigel-coated membranes, demonstrated that cells in the Cal27-NEK2 group exhibited significantly greater cell migration rates as well as higher numbers of migratory and invasive cells compared to those in the Cal27-vector group (Fig. 2C, D, E). In our previous studies, through IHC and qRT-PCR, we found that compared with normal oral mucosa, the expression of E-Cadherin was decreased, while the expressions of N-Cadherin and Vimentin were increased in OSCC tissues^14^. Thus, we hypothesized that NEK2 might promote the metastasis of OSCC by regulating EMT. To verify our hypothesis, we used qRT-PCR and western blotting to detect the EMT markers in Cal27-vector and Cal27-NEK2 stable cell lines. The results demonstrated that the ectopic expression of NEK2 decreased the expression of E-cadherin and increased the expressions of N-cadherin, Vimentin, and Slug (Fig. 2F, G). This is consistent with our previous findings, suggesting that NEK2 likely promotes the migration and invasion of OSCC cells by regulating the EMT process.

### The knockdown of NEK2 inhibits the migration and invasion of OSCC cells in vitro

**Fig. 3:**
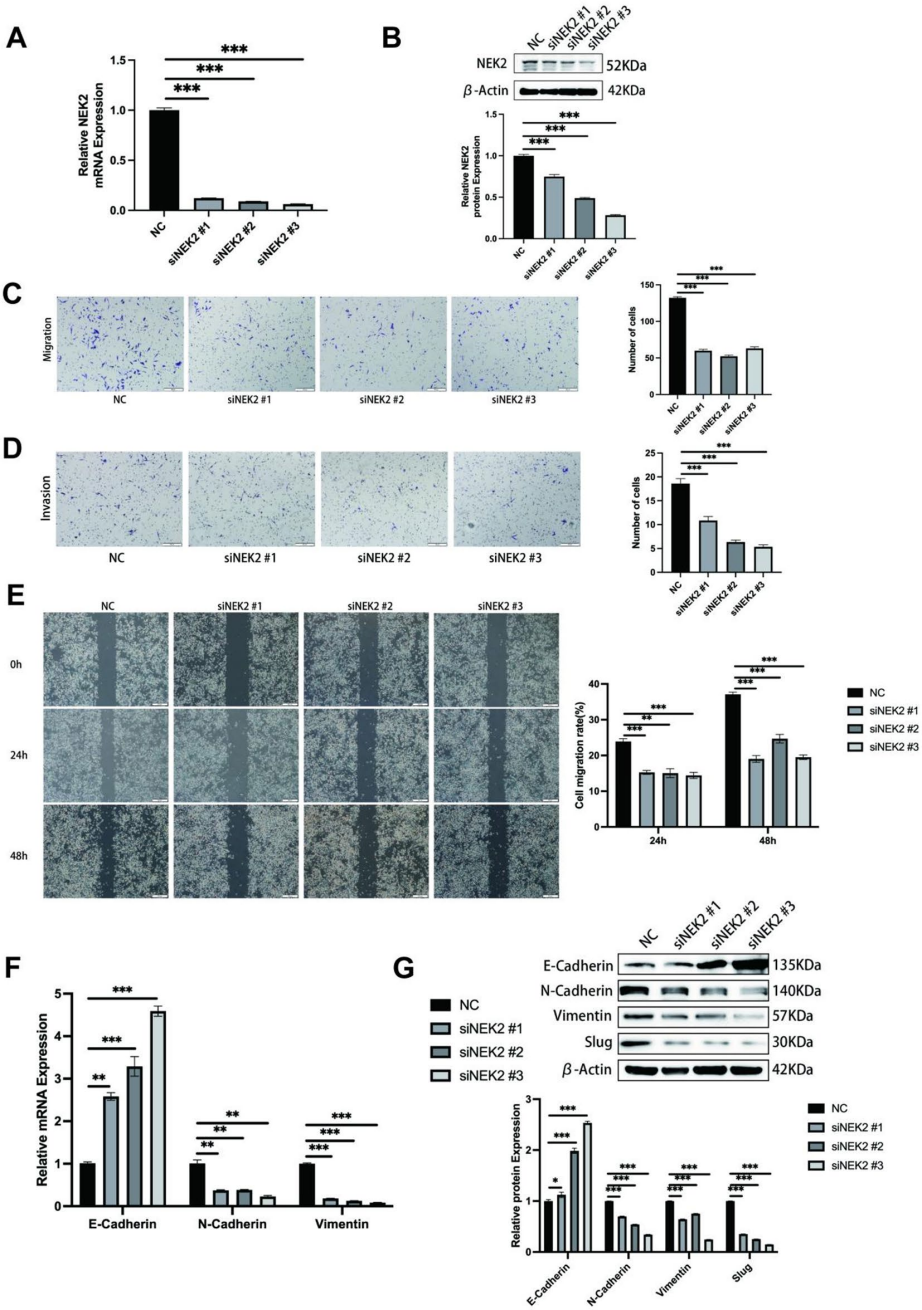
The knockdown of NEK2 inhibits the migration and invasion of OSCC cells in vitro. (**A**, **B**) The relative mRNA and protein expression levels of NEK2 in the SCC4 cell lines with and without NEK2 knockdown were determined by qRT-PCR (**A**) and western blotting (**B**), respectively. GAPDH and β-Actin served as loading controls for mRNA and protein detection, respectively. (**C**, ** D**) Cell migration assay (**C**) and invasion assay (**D**) were carried out in the indicated transfected cell lines. The images on the left are representative depictions, while the images on the right present the statistical outcomes. (**E**) The wound healing assay was performed in the indicated transfected cell lines. The left-hand images are representative illustrations, and the right-hand images show the statistical results. (**F**, ** G**) The relative mRNA and protein expression levels of EMT-associated factors were measured by qRT-PCR (**F**) and western blotting (**G**), respectively, in the indicated transfected cell lines. GAPDH and β-Actin served as loading controls for mRNA and protein detection, respectively. All data represent mean ± SD from three independent experiments. Student’s t-test, *P < 0.05, **P < 0.01, ***P < 0.001.

To investigate the impact of NEK2 downregulation on the invasive and migratory capabilities of OSCC cells, we performed transient transfection of three independent siRNAs to effectively knockdown NEK2 in SCC4 cells, which exhibit high endogenous NEK2 expression. Successful knockdown was confirmed at both transcriptional and protein levels by qRT-PCR and western blot analysis, respectively (Fig. 3A, B). The results of the wound healing assay and Transwell assay demonstrated that, compared with the negative control (NC) cells, SCC4 cells with NEK2 gene knockdown exhibited both reduced cell migration rates and decreased numbers of migratory and invasive cells (Fig. 3C, D, E). We further examined whether NEK2 silencing modulates EMT in SCC4 cells. The expression levels of EMT-related markers by qRT-PCR and western blot analysis revealed that NEK2 knockdown elevated the expression of E-cadherin, while reducing the expressions of N-cadherin, Vimentin, and Slug (Fig. 3F, G). The findings demonstrate that knockdown of NEK2 inhibits the migratory and invasive abilities of OSCC cells in vitro and simultaneously triggers a reversal of the EMT process.

### Overexpression of NEK2 promotes the growth and metastasis of OSCC in vivo

**Fig. 4:**
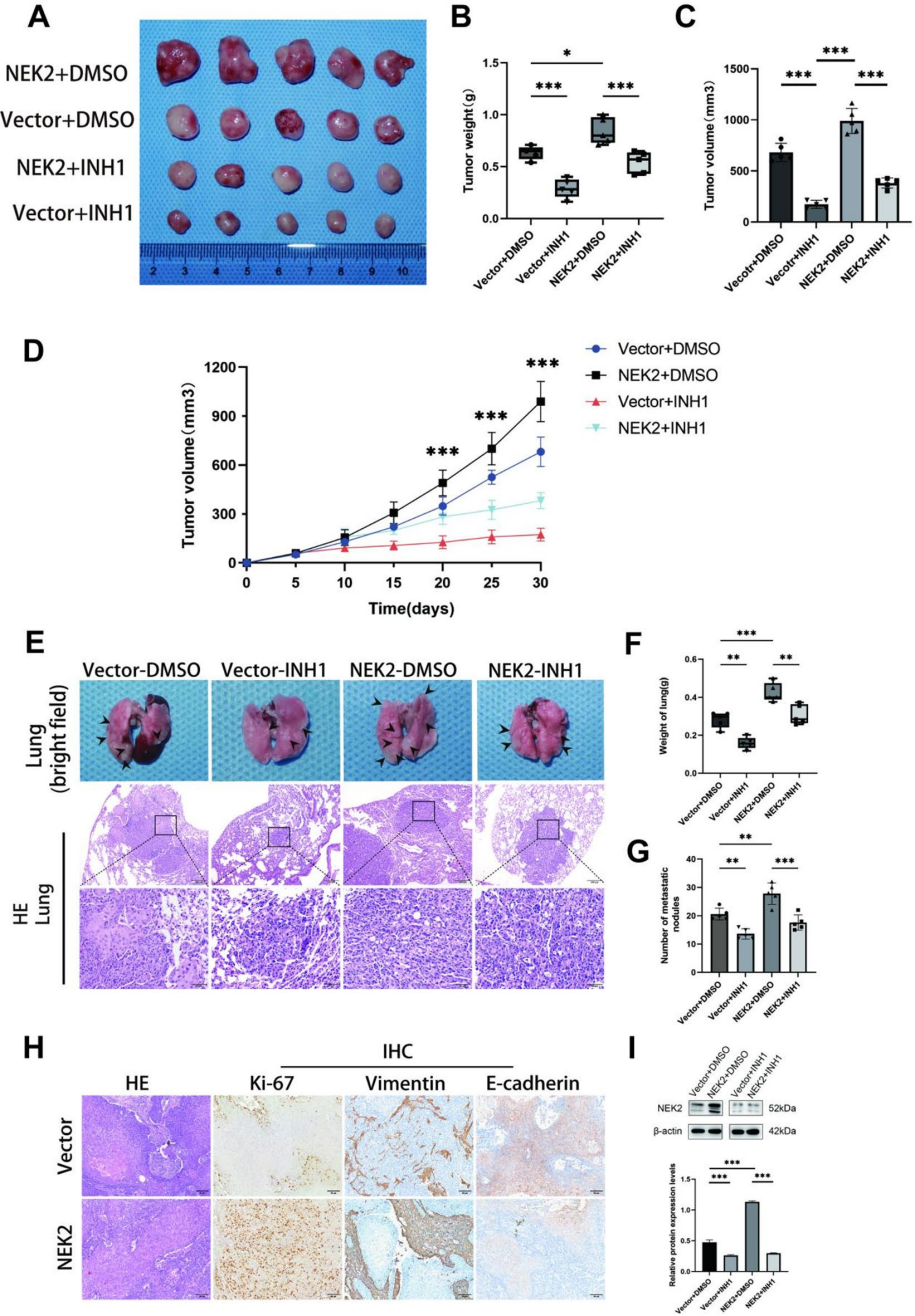
Overexpression of NEK2 promotes the growth and metastasis of OSCC in vivo and is inhibited by INH1. (**A**–**D**) Tumor photos of nude mice subcutaneously injected with Cal27-vector cells or Cal27-NEK2 cells after treatment with the vehicle (DMSO) or INH1 (**A**). The right image shows the tumor weight (**B**) and volume (**C**). The lower image shows the tumor growth curve (**D**). Data are presented as the mean ± SD of three independent experiments. Student’s t-test, *P < 0.05, ***P < 0.001. (**E**–**G**) Gross morphology of lungs from experimental metastasis model after treatment with the vehicle (DMSO) or INH1 (above **E**), and representative images of lung tumors stained with HE (below **E**, scale bars, 200 μm and 50 μm, respectively.). The right image shows the lung weight (**F**) and the number of lung tumors (**G**). Data are presented as the mean ± SD of three independent experiments. Student’s t-test, **P < 0.01, ***P < 0.001. (**H**) Representative images of subcutaneously xenografted tumors in mice stained by H&E, Ki−67, and IHC for EMT-related factors (E-cadherin, Vimentin). Scale bars, 50 μm. (**I**) The protein level of NEK2 in subcutaneously xenografted tumors of mice after treatment with the vehicle (DMSO) or INH1 was determined by western blotting. β-Actin was used as a loading control. Data are presented as the mean ± SD of three independent experiments. Student’s t-test, ***P < 0.001.

To evaluate the impact of NEK2 on the growth and metastasis of OSCC in vivo, we established subcutaneous xenograft tumor model by subcutaneously injecting either vector-control (Cal27-vector) or NEK2-overexpressing (Cal27-NEK2) stable cells into BALB/c nude mice. Measurements of tumor weight revealed that, compared with the Vector+DMSO group (0.63 ± 0.06 g), the NEK2 + DMSO group (0.84 ± 0.13 g) exhibited more pronounced subcutaneous tumor growth (Fig. 4A, B). Measurements of tumor volume revealed that, compared with the Vector+DMSO group (681.2 ± 89.8 mm^3^), the NEK2 + DMSO group (989.5 ± 122.9 mm^3^) exhibited more pronounced subcutaneous tumor growth (Fig. 4A, C). The tumor growth curves further indicated that the subcutaneous tumors in the NEK2 + DMSO group grew at a faster rate (Fig. 4D). Subsequently, to assess the impact of NEK2 on the distant colonization ability of OSCC cells, we established an experimental metastasis model by injecting equal numbers of stable Cal27-vector or Cal27-NEK2 cells into the lateral tail veins of BALB/c nude mice. The mice were sacrificed nine weeks post-injection, and lung tissues were collected for systematic analysis. Through lung tissue weighing, serial sectioning, H&E staining, and pathological examination, we identified and counted all microscopically detectable metastatic foci. As shown in the results, compared to the Vector+DMSO group, the NEK2 + DMSO group developed a greater number of metastatic nodules in the lung tissues (Fig. 4E, F,G). Collectively, these experimental results demonstrate that overexpression of NEK2 significantly promotes the growth of subcutaneous xenograft tumors of OSCC and enhances their ability to form metastatic foci in the lungs via hematogenous metastasis.

To investigate the mechanism by which NEK2 promotes OSCC metastasis, we performed IHC analysis on subcutaneous xenograft tumor tissues. The results showed that, compared with the Vector+DMSO group, the NEK2 + DMSO group exhibited significantly downregulated expression of the epithelial marker E-cadherin, while the expression of the proliferation marker Ki−67 and the mesenchymal marker Vimentin were both significantly upregulated in tumor tissues (Fig. 4H). Combined with the results from in vitro experiments demonstrating NEK2-mediated regulation of EMT-related proteins, these in vivo data further support the conclusion that NEK2 can reshape the phenotype of OSCC cells both in vitro and in vivo, and its metastasis-promoting effect may be closely associated with the induction of EMT.

### INH1 suppresses the NEK2-induced promotion of tumor growth and metastasis in vivo

Subsequently, to evaluate the potential clinical utility of targeting NEK2, we investigated the efficacy of INH1, a specific NEK2 inhibitor, against OSCC tumor growth and metastasis in BALB/c nude mice.

In the subcutaneous xenograft model, treatment with INH1 markedly inhibited tumor growth. Western blot analysis confirmed that NEK2 protein levels in the harvested OSCC tissues were substantially decreased upon INH1 treatment (Fig. 4I), verifying target engagement in vivo. Compared to the vehicle-treated mice, INH1 significantly reduced both tumor volume and mass in mice bearing either Cal27-vector or Cal27-NEK2 cells (Fig. 4A, B, C).

In the experimental lung metastasis model, INH1 treatment dramatically suppressed the metastatic burden. Mice treated with INH1 exhibited a significant reduction in the number of lung metastatic nodules (indicated by black arrows) and lower lung weight compared to the vehicle-treated mice (Fig. 4E, F, G). Histological examination by H&E staining further corroborated the decrease in pulmonary metastatic colonies in the INH1-treated group (Fig. 4E).

These results demonstrate that pharmacological inhibition of NEK2 by INH1 effectively suppresses OSCC tumor growth and metastatic colonization in the lungs in vivo.

### Identification of signaling pathways regulated by NEK2 in OSCC

To elucidate the downstream pathways mediated by NEK2, we performed Gene Ontology (GO) and Kyoto Encyclopedia of Genes and Genomes (KEGG) enrichment analyses on differentially expressed genes between NEK2-overexpressing and vector control Cal27 cells.

**Fig. 5:**
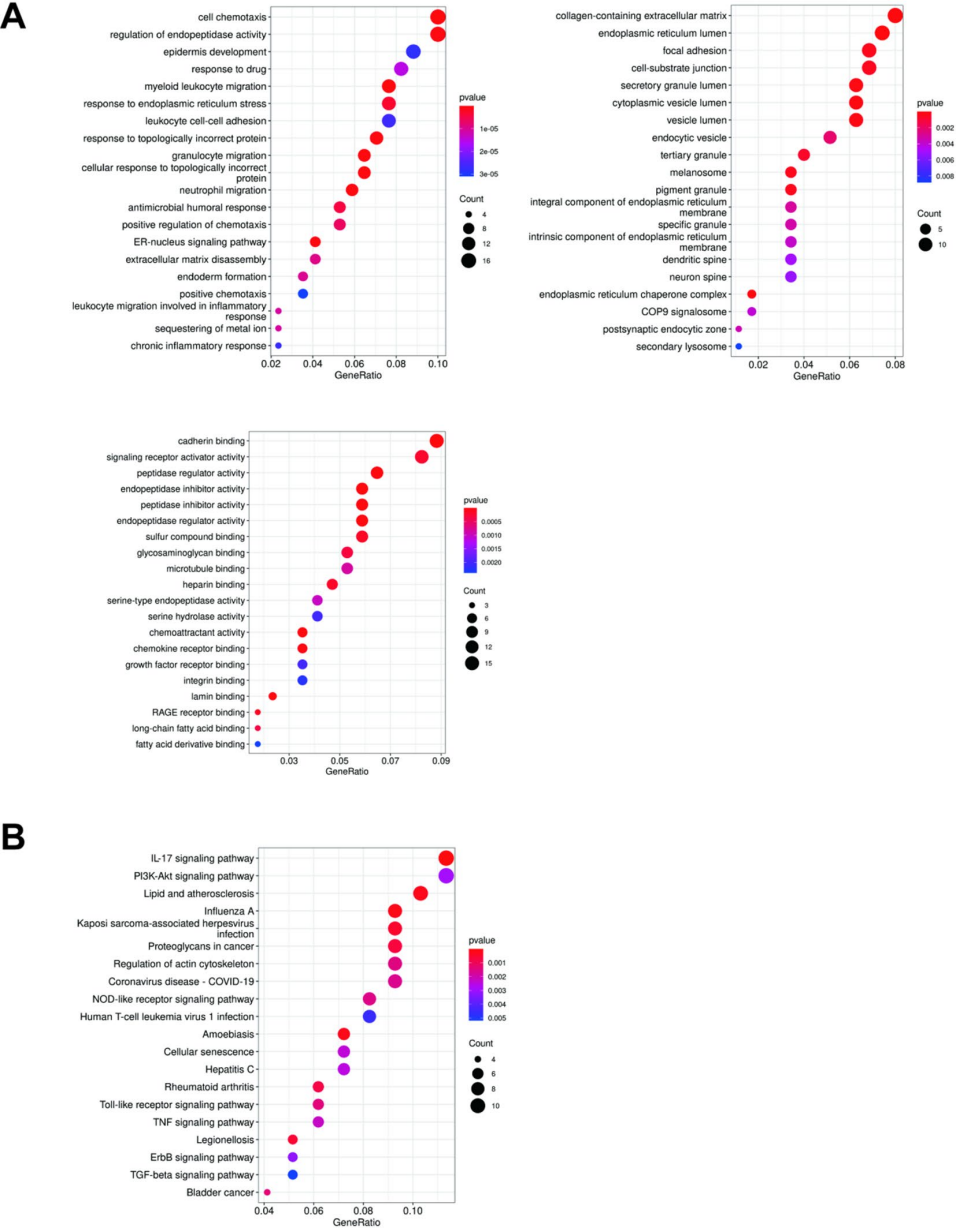
Characteristics of co-expressed genes associated with NEK2 in OSCC. (**A**) GO analysis. The left image shows GO-BP, the right image shows GO-CC, and the lower image shows GO-MF. (**B**) Results of KEGG pathway enrichment analysis.

GO analysis revealed that NEK2-upregulated genes were significantly enriched in biological processes (BP) related to cell chemotaxis and regulation of peptidase activity. At the cellular component (CC) level, these genes were associated with the collagen-containing extracellular matrix, endoplasmic reticulum lumen, and focal adhesions. Their molecular functions (MF) were linked to cadherin binding, signaling receptor activator activity, and peptidase regulation activity (Fig. 5A).

KEGG pathway analysis further indicated that NEK2 overexpression prominently activated the IL-17 signaling pathway and the PI3K-Akt signaling pathway (Fig. 5B).

These findings suggest that NEK2 may promote OSCC progression by modulating pathways involved in immune response, cell survival, and extracellular matrix remodeling, which are processes closely associated with tumor invasion and metastasis.

**Fig. 6:**
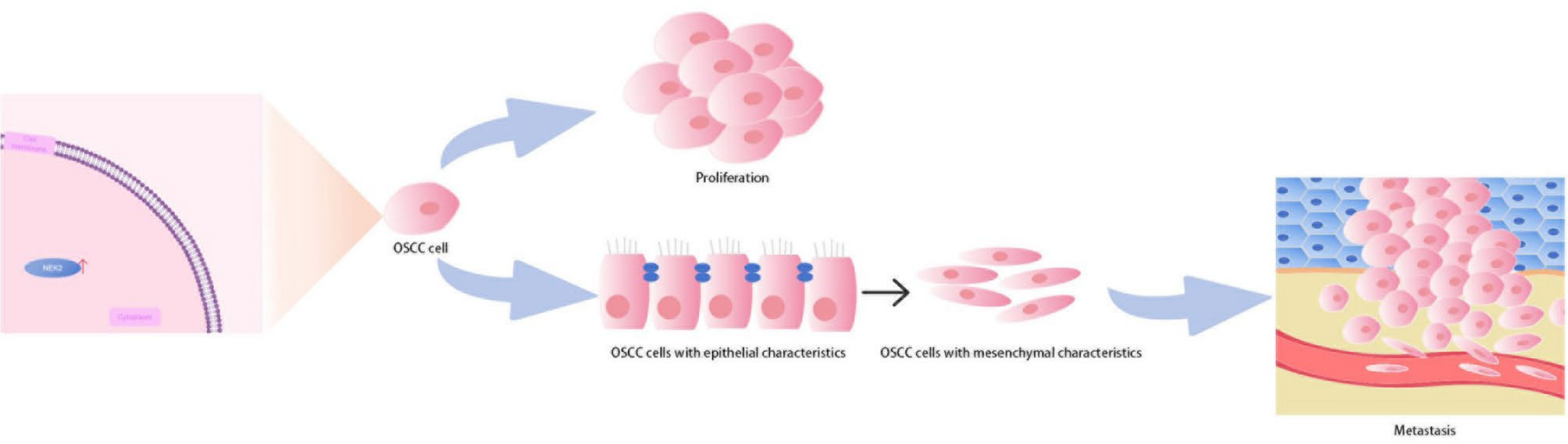
Schematic diagram of the oncogenic role of NEK2 in OSCC.

## Discussion

OSCC represents the most prevalent malignant neoplasm in the oral and maxillofacial region. While previous studies have demonstrated that NEK2 is overexpressed in OSCC tissues^27^, its functional significance in OSCC remains to be elucidated. This study was the first to systematically elucidate the oncogenic role of NEK2 in OSCC. We found that NEK2 is significantly overexpressed in both OSCC tissues and cell lines, and its high expression is closely associated with poor patient prognosis. Overexpression of NEK2 promoted the migration and invasion of OSCC cells in vitro, as well as tumor growth and metastasis in vivo. NEK2 induced the EMT program, and likely mediated the promotion of distant OSCC metastasis by regulating EMT. Furthermore, NEK2 overexpression activates signaling pathways such as IL-17 and PI3K-Akt. Targeted inhibition of NEK2 using INH1 effectively suppresses the growth and lung metastasis of OSCC in vivo. Collectively, these findings establish NEK2 as a key driver in the progression of OSCC (Fig. 6).

NEK2, a key serine/threonine kinase involved in cell cycle regulation, serves as a core regulator of centrosome formation, proper assembly, and interphase maintenance during mitosis^31^. In recent years, the role of NEK2 in tumorigenesis and progression has garnered increasing attention. Studies have shown that NEK2 is upregulated at the mRNA and/or protein levels in various malignancies and cell lines, including osteosarcoma, lung adenocarcinoma, esophageal squamous cell carcinoma, and head and neck squamous cell carcinoma^32–35^. In this study, we similarly observed significant upregulation of NEK2 expression in both clinical OSCC tissues and human-derived cell lines, suggesting that its aberrant expression may be closely associated with OSCC progression. Furthermore, the expression level of NEK2 is strongly correlated with patient prognosis in multiple cancers, with high NEK2 expression often indicating poor survival outcomes^36–38^. Our findings further confirm that elevated NEK2 expression is significantly associated with unfavorable prognosis in OSCC patients, indicating that assessing NEK2 expression holds important reference value for evaluating the prognosis of OSCC patients.

Recent studies have further revealed that NEK2 promoted tumorigenesis through diverse mechanisms, such as targeting the cell cycle^39^, remodeling the extracellular matrix^40^, activating ATG5-mediated autophagy^41^, and promoting TP53 ubiquitination^23^. This study was the first to validate the tumor-promoting function of NEK2 in the OSCC system: overexpression of NEK2 enhanced the migration and invasion of OSCC cells in vitro, induced EMT, and promoted tumor growth and metastasis in vivo. It was noteworthy that EMT, as a core mechanism of tumor invasion and metastasis, played a key role in OSCC progression^42,43^. Evidence indicated that NEK2 was closely associated with EMT in tumors, where it promoted tumor metastasis and invasion by regulating the EMT process in malignant cells^44–46^. Our previous work found that NEK2 expression was closely related to changes in EMT markers. This study further confirmed through functional experiments that overexpression of NEK2 downregulated E-cadherin and upregulated N-cadherin, Vimentin, and Slug, whereas knockdown of NEK2 produced the opposite molecular phenotype. These results suggested that NEK2 likely promoted OSCC invasion and metastasis by regulating the EMT program, providing an important direction for investigating its tumor-promoting mechanisms.

Transcriptomic analysis in this study revealed that NEK2 activated two key signaling pathways, IL−17 and PI3K-Akt, to drive OSCC progression. The IL−17 pathway was traditionally recognized as a central pathway in inflammation and autoimmune diseases^47^. Recent studies indicated that this pathway also played an important role in the tumor microenvironment, where it could directly promote the proliferation of precancerous cells by inducing mitotic signaling and accelerate tumor growth while enhancing therapy resistance by remodeling the tumor stromal architecture^48^. The PI3K-Akt pathway, a classic pro-proliferation and survival pathway, was aberrantly activated in various cancers. Previous research suggested that NEK2 could promote the malignant progression of hepatocellular carcinoma by activating the PI3K/AKT-NFκB signaling axis, thereby driving tumor proliferation, metastasis, and chemoresistance^49^. Combined with the findings of this study, it was plausible that NEK2 exacerbated the pathogenesis and development of OSCC by activating the IL−17 and PI3K-Akt signaling pathways.

Studies have shown that targeting NEK2, either alone or in combination with other therapies, represented a promising therapeutic strategy^36,50^. Several specific NEK2 inhibitors have been developed to date, and multiple candidates have demonstrated encouraging antitumor efficacy in both in vitro and in vivo models^51–54^. For instance, INH1, a dual-target NEK2 inhibitor, was a highly specific small-molecule compound that targets the Hec1/Nek2 interaction. It exerted its antitumor effect by directly binding to Hec1 and disrupting the Hec1/Nek2 complex. This interaction led to a significant reduction in NEK2 protein levels, subsequently causing misalignment of mitotic chromosomes, spindle defects, and ultimately, apoptosis. In xenograft tumor models, INH1 treatment has shown significant tumor growth inhibition without inducing apparent systemic toxicity^55^. Our study confirmed that in vivo, INH1 not only inhibited the growth of primary tumors but also significantly reduced the formation of lung metastatic foci—a finding of considerable importance. Given the limited treatment options and poor prognosis for OSCC patients, particularly those with advanced, recurrent, or metastatic disease, targeting NEK2 might offer a novel adjunctive therapeutic strategy for this patient population. Our research provided a solid preclinical foundation for the development of NEK2 inhibitors and explores their potential use in combination with existing radiotherapy, chemotherapy, or immunotherapy.

Of course, this study also had some limitations. First, the specific molecular interfaces through which NEK2 regulates the IL−17 and PI3K-Akt pathways remained incompletely elucidated. Second, although INH1 had demonstrated efficacy in animal models, its pharmacokinetics, toxicity profile, and effectiveness in humans required further evaluation.

In summary, this study systematically revealed a novel mechanism through which NEK2 drived the growth and metastasis of OSCC. These findings not only deepened the understanding of the molecular mechanisms underlying OSCC proliferation, invasion, and metastasis but also established NEK2 as a promising prognostic biomarker and therapeutic target, providing new theoretical foundations and strategic directions for the precision treatment of OSCC.

## Supplementary Information

Below is the link to the electronic supplementary material.

Supplementary Material 1

## Data Availability

The datasets used and/or analyzed during the current study are available from the corresponding author on reasonable request.
