# Supplementary figures and images for: NEK2 promotes oral squamous cell carcinoma progression and serves as a diagnostic and therapeutic target
Source: Sci Rep. 2026 Apr 10;16:16116. doi: 10.1038/s41598-026-47174-6 (PMC13199490; doi:10.1038/s41598-026-47174-6)

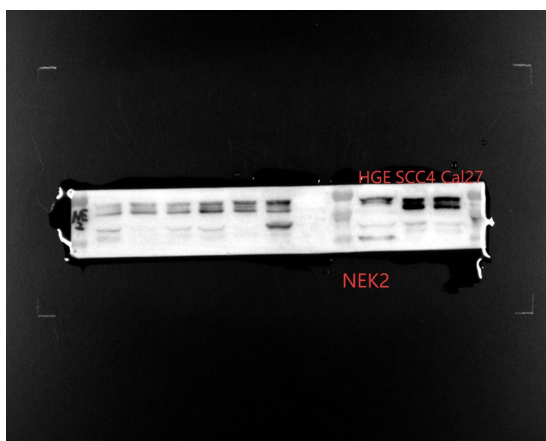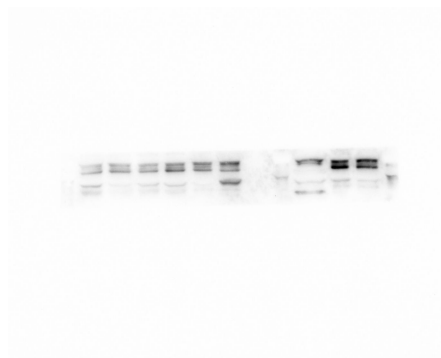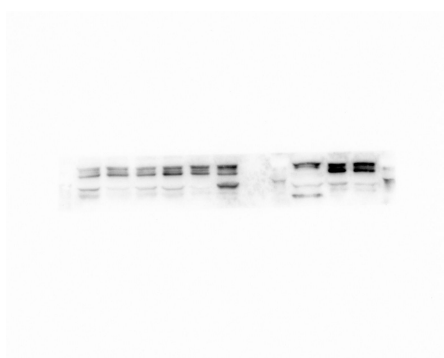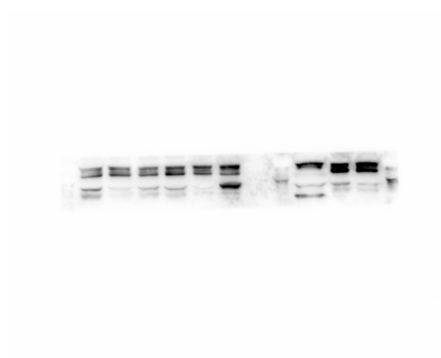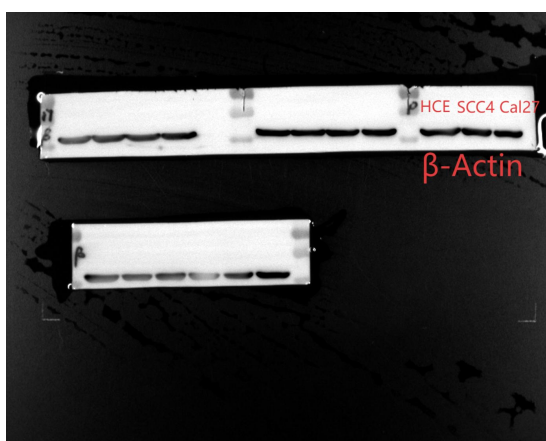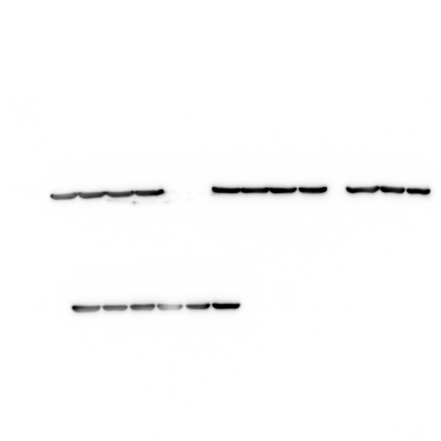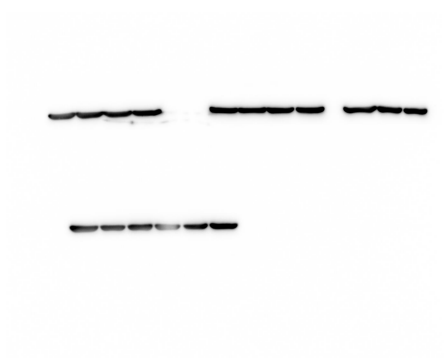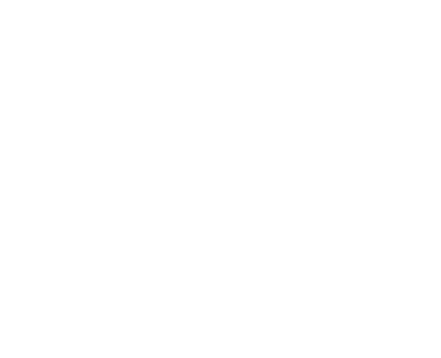

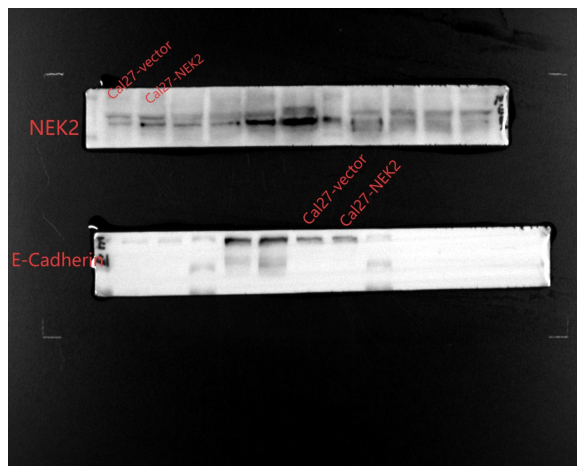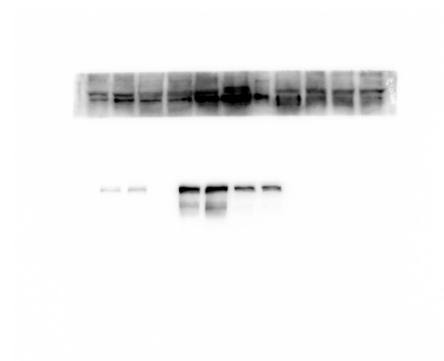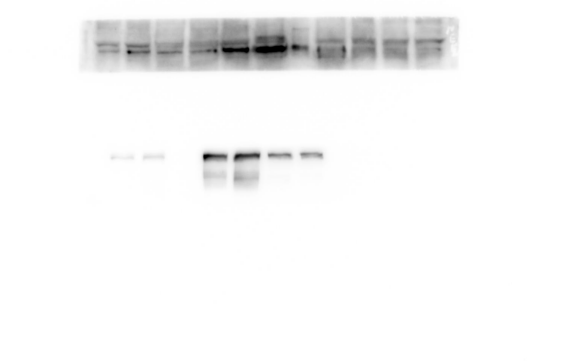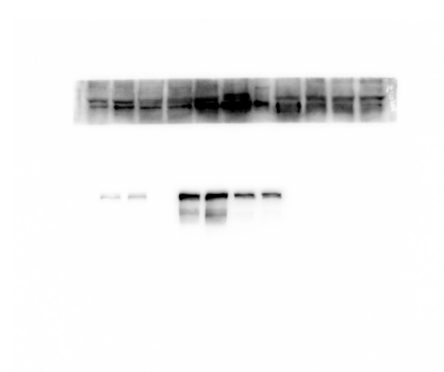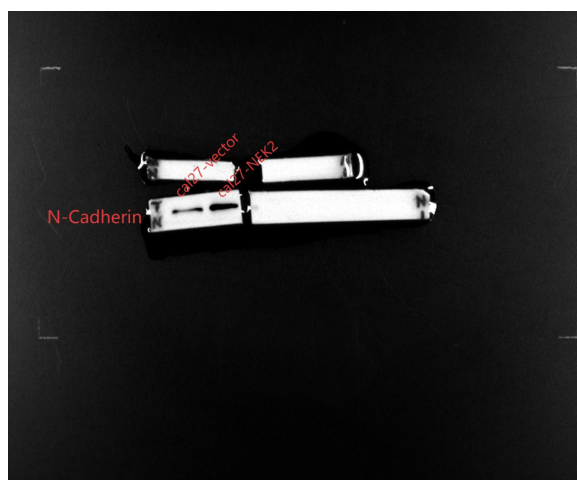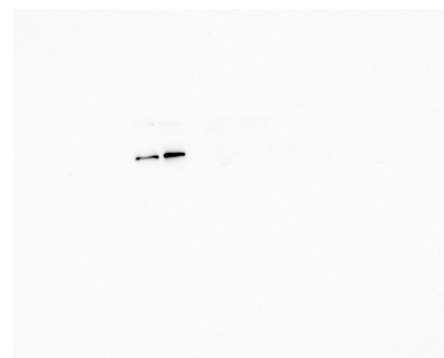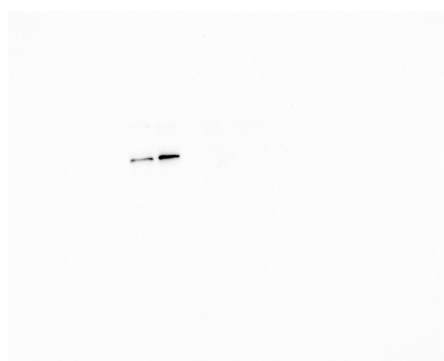

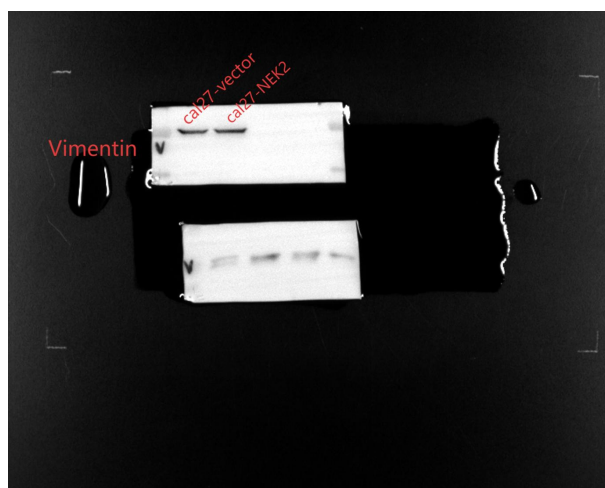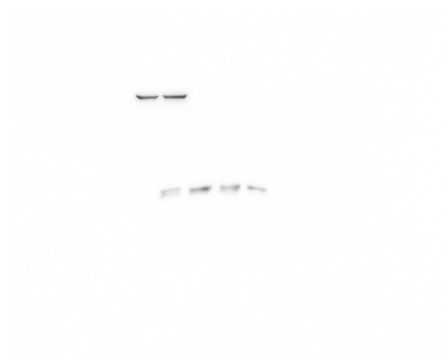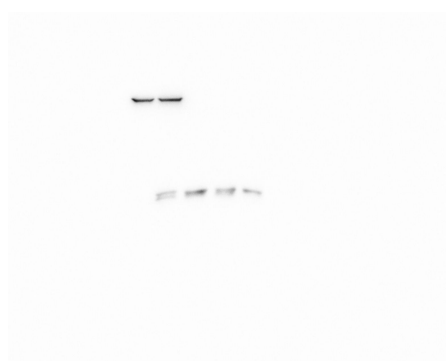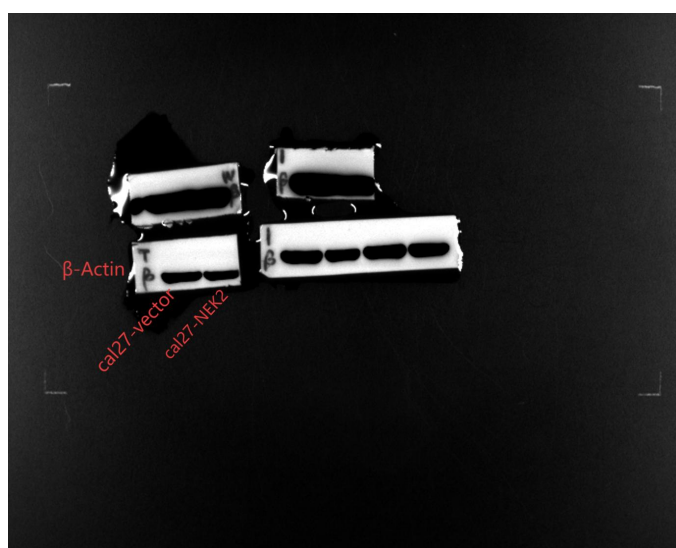

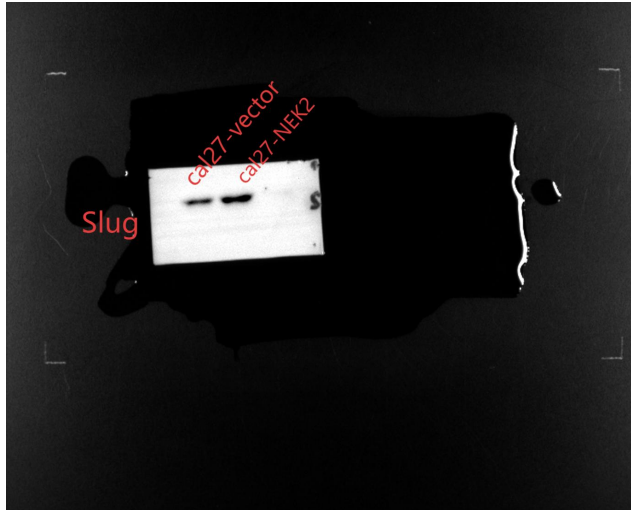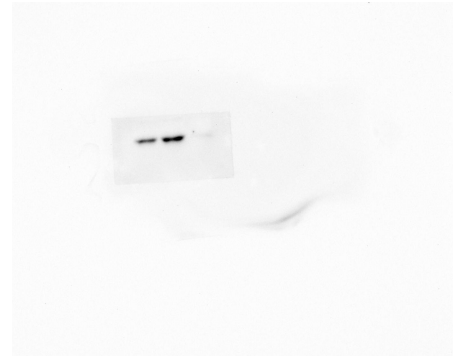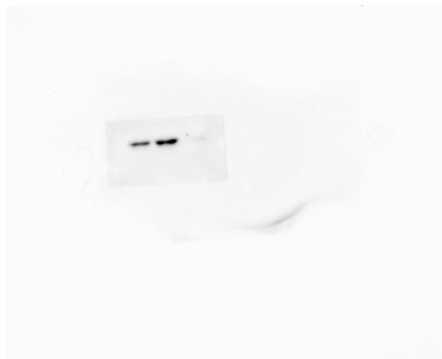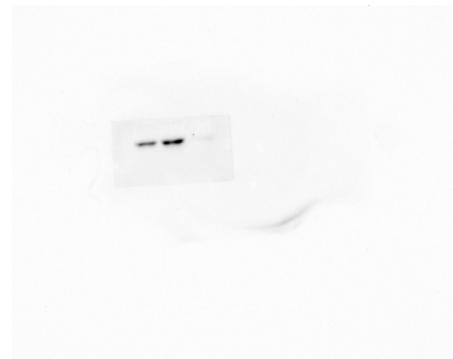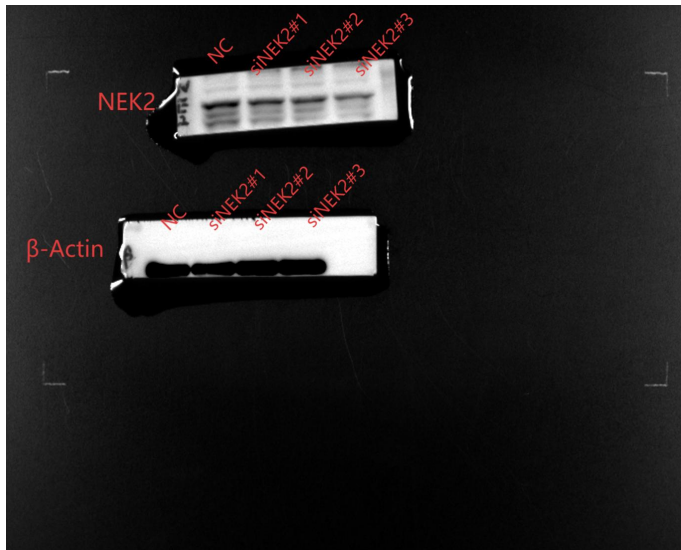

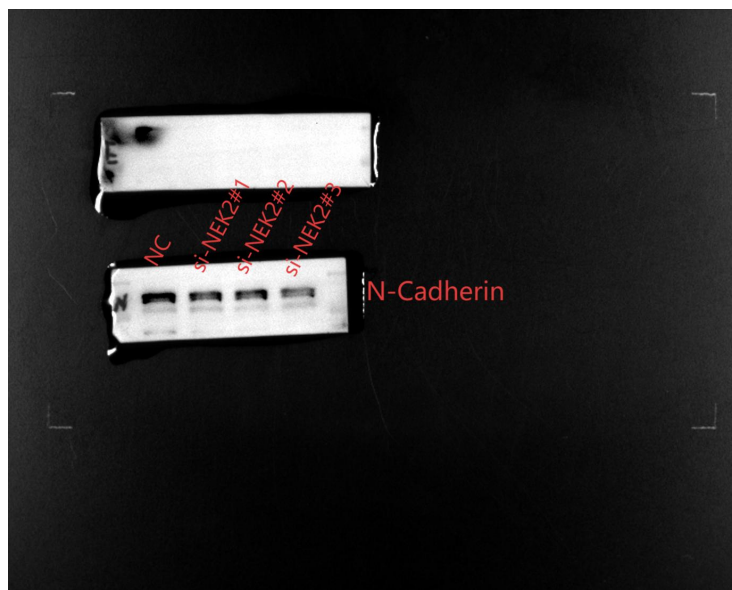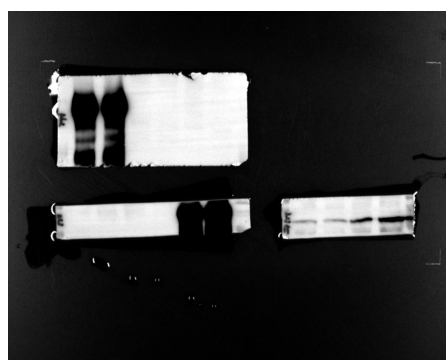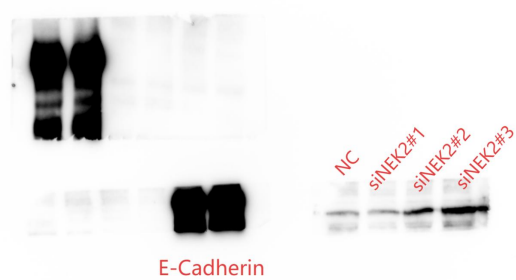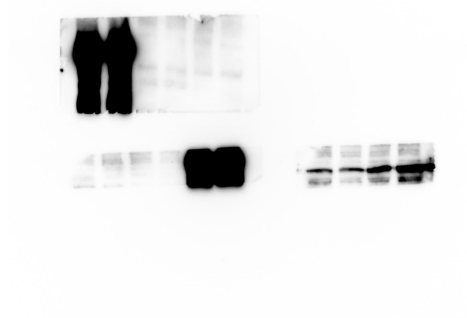

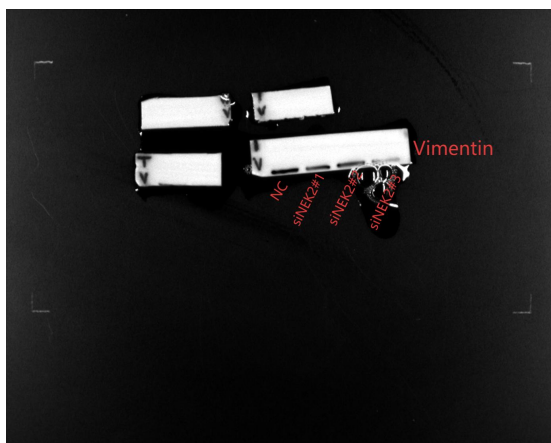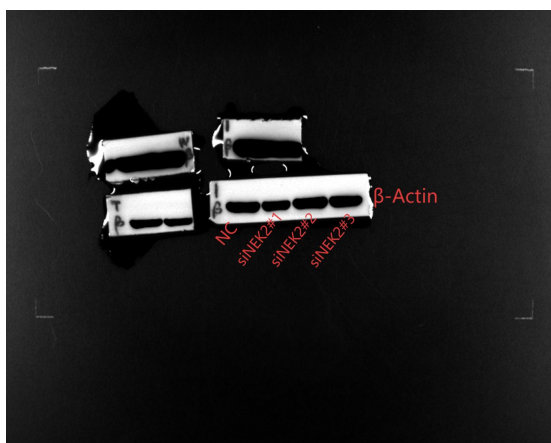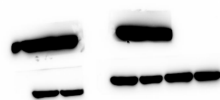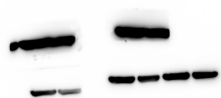

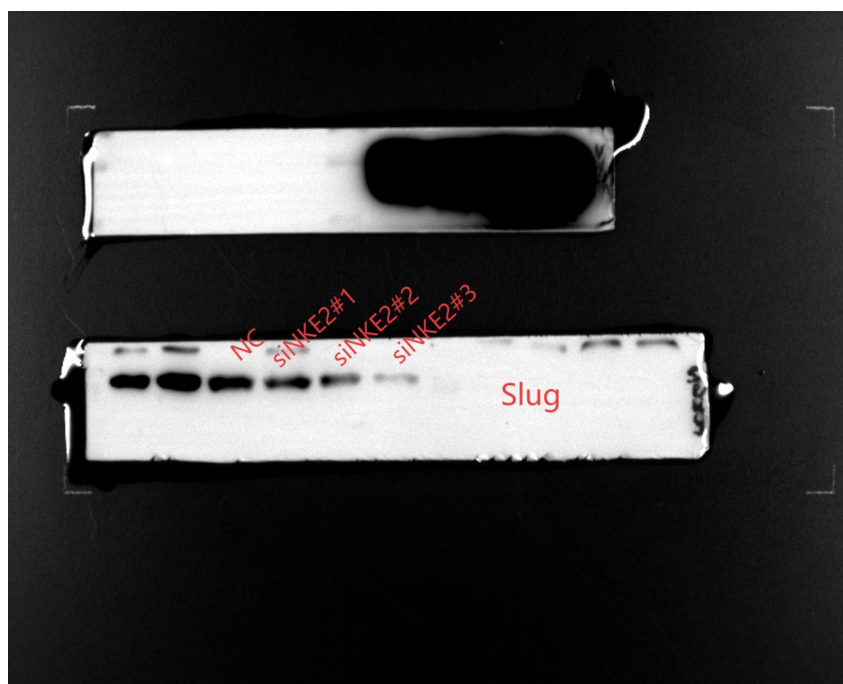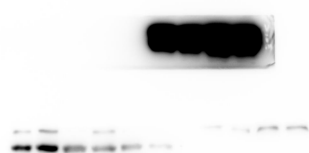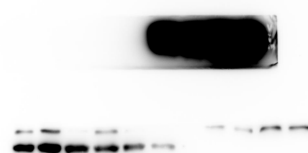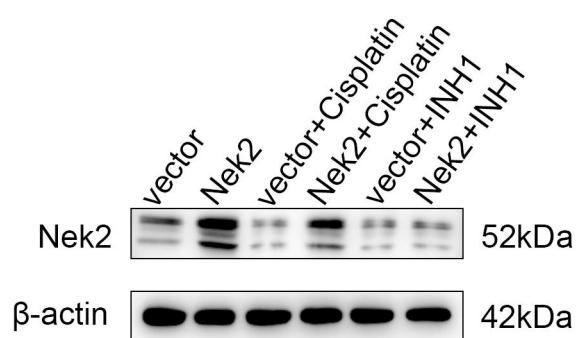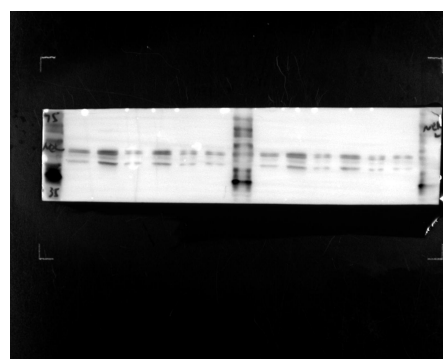

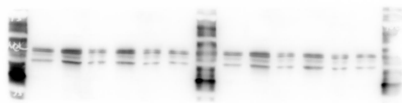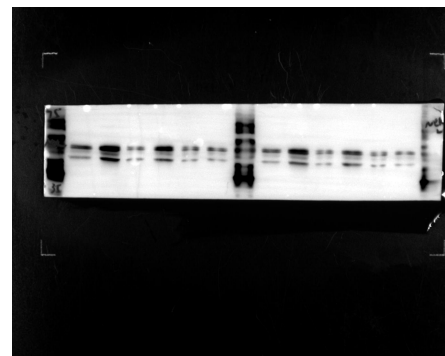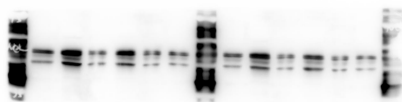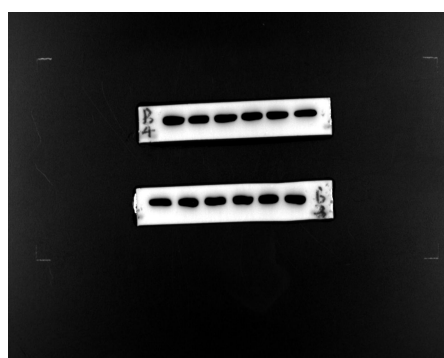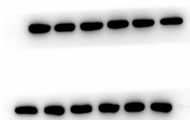

Supplement: Supplementary file 1 — Supplementary Material 1 [file 41598_2026_47174_MOESM1_ESM.pdf]
